# Prevalence of colorectal cancer and its precursor lesions in symptomatic patients under 55 years of age undergoing total colonoscopy: results of a large retrospective, multicenter, controlled endoscopy study

**DOI:** 10.1007/s00384-021-03898-7

**Published:** 2021-03-05

**Authors:** Katharina Stratmann, Katarzyna Czerwinska, Natalie Filmann, Wolfgang Tacke, Christoph Weber, Herbert Bock, Irina Blumenstein

**Affiliations:** 1 Department of Medicine I, J.W. Goethe University Frankfurt, Frankfurt, Germany; 2 Institute of Biostatistics and Math Modeling, J.W. Goethe University Hospital Frankfurt, Frankfurt, Germany; 3 Facharztzentrum am Taunus, Taunus, Germany; 4 Gastroenterologische Facharztpraxis, Zeil, 65 Frankfurt, Germany

**Keywords:** Colorectal cancer, Lesions, Total colonoscopy

## Abstract

**Purpose:**

Colorectal cancer (CRC) is the second most common cancer in Germany. Around 60,000 people were diagnosed CRC in 2016 in Germany. Since 2019, screening colonoscopies are offered in Germany for men by the age of 50 and for women by the age of 55. It is recently discussed if women should also undergo a screening colonoscopy by the age of 50 and if there are any predictors for getting CRC.

**Methods:**

Colonoscopies of 1553 symptomatic patients younger than 55 years were compared with colonoscopies of 1075 symptomatic patients older than 55 years. We analyzed if there are any significant differences between those two groups in the prevalence of CRC and its precursor lesions or between symptomatic men and women. We evaluated if there is a correlation between abdominal symptoms and the prevalence of CRC.

**Results:**

In 164/1553 symptomatic patients, 194 (12.5%) polyps were detected. In total, six colorectal carcinomas (0.4%) were detected. There were no significant differences between men and women. In symptomatic patients ≥ 55 years, significantly more polyps were found (*p*<0.0001; 26.6% vs. 12.5%). Totally, 286 polyps (26.6%) were removed in 1075 symptomatic patients older than 55 years. Anorectal bleeding was the only abdominal symptom being a significant indicator for the prevalence of the occurrence of colon and rectum cancer in both groups (*p*=0.03, OR=2.73 95%-CI [1.11;6.70]), but with only low sensitivity (44%).

**Conclusion:**

Due to no significant differences in men and women, we recommend screening colonoscopies also for women by the age of 50.

## Introduction and background

The colorectal cancer (CRC) is the second most common cancer in Germany. Every eighth cancer disease is a colon or a rectum cancer [1]. Around 60,000 people (around 32 000 men and 26 000 women) were diagnosed colorectal cancer in 2016 in Germany [1]. In Europe, the incidence of colorectal cancer is about 20–70/100,000 residents [2].

It is known that colonoscopies are an effective tool for screening colorectal neoplasms or its precursor lesions [3–7]. In 2002, Germany was the first country worldwide which offered screening colonoscopies for all citizens 55 years of age and older [8–10]. Since 2019, in Germany, it is recommended that men should undergo a screening colonoscopy by the age of 50 due to a higher risk for CRC in men compared with women, while women should still do a screening colonoscopy by the age of 55 [11, 12].

There is only little data for any clinical predictors—besides, i.e., age, gender, or family history—for getting colorectal cancer.

The aim of our study was to evaluate if patients younger than 55 years suffering under specific abdominal symptoms have a higher risk of getting colorectal cancer than patients older than 55 years or if there are significant differences between those two groups. Furthermore, we investigated if men with specific abdominal symptoms have significant more findings (i.e., polyps or CRC) during a colonoscopy than women.

Additionally, we were interested if there are any abdominal symptoms which might be a predictor for the occurrence of polyps or even CRC.

Due to the fact men are supposed to have a higher risk for getting CRC and for having more precursor lesions than women, we hypothesize there may be a significant difference between men and women in finding a CRC or precursor lesions.

## Methods

### Patient cohort

Participants who were eligible for the study were symptomatic patients between 18 and 54 years old living in Hesse—one of the sixteen federal states in Germany with about 6 million inhabitants. These participants underwent a colonoscopy in an outpatient clinic. Only patients having their first colonoscopy were considered for this study. We evaluated patients with the following symptoms: abdominal cramps (i), abdominal pain (ii), altered bowel habits (iii), anorectal bleeding (iv), diarrhea (v), anemia (vi), weight loss (vii), occult bleeding (verified by a positive fecal occult blood test) (viii), melena (ix), and constipation (x).

The patients eligible for the study provided written informed consent.

Data were collected prospectively from 1 October 2008 until 30 September 2010 in the context of an integrated care program. The colonoscopies were performed by gastroenterologists in 49 different outpatient clinics in Hesse.

The control group included symptomatic patients of at least 55 years of age from the same Hessian screening population. The colonoscopies were done by the same group of gastroenterologists. This control cohort was already examined in detail in a previous study [13].

We excluded patients having already had a colonoscopy before or having had a history of colorectal surgery. Also, patients with an increased risk for CRC (surveillance after resection of a CRC, surveillance after polypectomies, known inflammatory bowel disease, familial adenomatous polyposis, hereditary nonpolyposis colon cancer, positive family history of CRC) were excluded. Only total colonoscopies were considered for this study. In total, our cohort included 2227 colonoscopies, but we had to exclude around 674 colonoscopies due to a lack of data.

### Study procedures

We examined retrospectively the number of colonoscopies in which tumor-suspicious lesions were found and removed. The findings were categorized into the following groups: polyps (confirmed by histology, number, location) and CRC. In most cases, the findings were removed immediately—either by snare or forceps polypectomy—during the procedure and were investigated by a pathologist afterward. CRC was confirmed by histology and removed via surgery.

Furthermore, we compared the symptomatic patients under 55 and over 55 years of age. We analyzed the data for tumor-suspicious lesions and if there are significant differences in the occurrence of tumor-suspicious lesions in patients younger than 55 and older than 55 years of age. Additionally, we looked for a correlation between symptoms and the occurrence of tumor-suspicious lesions.

The results of the symptomatic patients undergoing colonoscopies were transmitted to IOMTech, Berlin, Germany, a company for quality assurance and storage. IOMTech collected the data from established gastroenterologists. The data were collected prospectively. We were able to investigate the data retrospectively after receiving the ethical approval.

### Data analysis

Categorical variables were compared with the *χ*^2^ test, Fisher’s exact test, or the chi-square test of homogeneity.

All tests were two-tailed, and the level of significance was 5%. Statistical analysis was performed with R (version 4.0.2, R Foundation of Statistical Computing, Vienna, Austria) and BiAS (version 11.02, epsilon Verlag, Hochheim Darmstadt, Germany).

## Results

### Patient characteristics

**Fig. 1:**
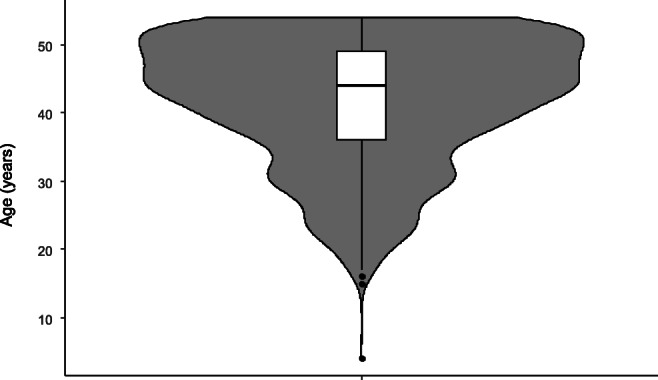
Age distribution of symptomatic patients

From 1 October 2008 to 30 September 2010, in 1553 symptomatic patients under 55 years of age, a total colonoscopy was performed. From 1553 patients, there were 864 female and 689 male patients. The age distribution is shown in Fig. 1. The median age in men was 44 years, in women 43 years.

### Indications for symptomatic colonoscopies

We evaluated patients having different abdominal symptoms. Symptoms as abdominal cramps (i), abdominal pain (ii), altered bowel habits (iii), anorectal bleeding (iv), diarrhea (v), anemia (vi), weight loss (vii), occult bleeding (verified by a positive fecal occult blood test) (viii), melena (ix), and constipation (x) were considered. In Table 1, indications for colonoscopies are presented.

**Table 1:** Indications for symptomatic colonoscopies

	Gender			
Symptoms	Symptomatic men (%)	Symptomatic women (%)	Total (%)	p
Abdominal cramps	5 (1)	7 (1)	12 (1)	n.s.
Abdominal pain	191 (28)	313 (36)	504 (32)	<0.0001
Altered bowel habits	31 (4)	30 (3)	61 (4)	n.s.
Anorectal bleeding	190 (28)	139 (16)	329 (21)	<0.0001
Diarrhea	52 (8)	83 (10)	135 (9)	n.s.
Anemia	4 (1)	21 (2)	25 (2)	0.004
Weight loss	4 (1)	4 (0)	8 (1)	n.s.
Not known (n.k.)	192 (28)	232 (27)	424 (27)	n.s.
Occult bleeding	8 (1)	9 (1)	17 (1)	n.s.
Melena	0 (0)	2 (0)	2 (0)	n.s.
Constipation	12 (2)	24 (3)	36 (2)	n.s.
Total	689 (100)	864 (100)	1553 (100)	

Totally, 748/1553 (48%) had unspecific symptoms as abdominal cramps, abdominal pain, altered bowel habits, diarrhea, or constipation. Three hundred eighty-one/1553 (25%) had symptoms like anorectal bleeding, anemia, weight loss, occult bleeding, or melena which are easier to objectify. In 424/1553 (27%), symptoms were not further specified.

### Prevalence of polyps and colorectal cancer in symptomatic patients < 55

In 164/1553 symptomatic patients, 194 (12.5%) polyps were detected. The median age of patients in which polyps were found and removed was 48 years (1st Quartile 43.3 years, 3rd Quartile 51.75 years).

In Table 2, the rate of removed polyps is shown.

**Table 2:** Removed polyps in symptomatic patients < 55 years

	Gender		
Polyps	Men (%)	Women (%)	Total (%)
No polyps found	592 (86)	767 (89)	1359 (88)
Removed polyps	97 (14)	97 (11)	194 (12)
Total	689 (100)	864 (100)	1553 (100)

*p* value 0.11

There was no significant difference in the appearance of findings and removed polyps between men and women aged < 55 years.

In total, 6/1553 (0.4%) patients had a colorectal cancer (5 colon cancer, 1 rectum cancer) which were found during the colonoscopy (see Table 3).

**Table 3:** Colorectal cancer in symptomatic patients < 55 years

	Gender		
Colorectal cancer	Men (%)	Women (%)	Total (%)
	688 (100)	859 (100)	1547 (100)
Colorectal cancer	1 (0)	5 (0)	6 (0)
Total	689 (100)	864 (100)	1553 (100)

*p* value >0.2

There are no significant differences between symptomatic men and women.

### Histological findings in symptomatic patients < 55 years

In our retrospective data analysis, results from 95 patients (out of 164 patients) from the histological refurbishment could be transmitted to us. In these 95 patients, 103 polyps were detected. The histological findings were distinguished in the following groups: adenomas with high-grade dysplasia (villous, tubulovillous, and tubular) (a), adenomas with low-grade dysplasia (villous, tubulovillous, and tubular) (b), traditional serrated adenomas low grade (c), sessile serrated adenoma low grade (d), hyperplastic polyps (e), and other benign polyps (f). The distribution of the histological findings is presented in Table 4.

**Table 4:** Findings in symptomatic patients < 55 years

	Gender		*p* value
Histology	w	m	
Adenomas with high-grade dysplasia (*n*=2)	2	0	0.16
Adenomas with low-grade dysplasia - villous - tubulovillous - tubular (*n*=46)	19 0 1 18	27 0 4 23	>0.2
Traditional serrated adenomas low-grade (*n*=2)	0	2	0.16
Sessile serrated adenomas low-grade (*n*=0)	0	0	
Hyperplastic polyps (*n*=39)	15	24	0.15
Other benign polyps (*n*=14)	9	5	>0.2

In 44.7% (46/103), adenomas with a low-grade dysplasia were found. Hyperplastic polyps were removed in 37.9% (39/103). Other benign polyps were detected in 13.6%. In only 1.9%, there were adenomas with a high-grade dysplasia. There were no significant differences in the findings between men and women.

### The screening population – comparison between symptomatic patients younger and older than 55 years of age

In the control group, there were 1075 (468 men and 607 women) patients undergoing a colonoscopy due to abdominal symptoms. No significant differences in gender distribution could be found in these two groups (*p*>0.2).

In symptomatic patients ≥ 55 years of age, significantly more polyps were found (*p*<0.0001; 26.6% vs. 12.5%). Totally, 286 polyps (26.6%) were removed in 1075 patients (Table 5).

**Table 5:** Removed polyps in symptomatic patients < and ≥ 55 years

	Age		
Polyps	<55 (%)	≥55 (%)	Total (%)
No polyps found	1359 (88)	789 (73)	2148 (82)
Removed polyps	194 (12)	286 (27)	480 (18)
Total	1553 (100)	1075 (100)	2628 (100)

*p* value <0.0001

Colorectal cancer was detected in 12 from 1075 symptomatic patients over the age of 55 years. In comparison with the patients younger than 55 years, there are significant more colorectal cancers in symptomatic patients > 55 years than to our screening cohort (Table 6).

**Table 6:** Colorectal cancer in symptomatic patients < and ≥ 55 years

	Age		
Colorectal cancer	<55 (%)	≥55 (%)	Total (%)
	1547 (100)	1063 (99)	2610 (100)
Colorectal cancer	6 (0)	12 (1)	18 (0)
Total	1553 (100)	1075 (100)	2628 (100)

*p* value 0.03

### Correlation between symptoms and occurrence of tumor-suspicious lesions

Anorectal bleeding was the only abdominal symptom which was a significant indicator for the prevalence of the occurrence of colon and rectum cancer in both groups (*p*=0.03, OR=2.73 95%-confidence interval (CI) [1.11;6.70]), but with only low sensitivity (44%).

## Discussion

Screening colonoscopies are used for the detection and prevention of colorectal cancer in asymptomatic patients [14]. There are hardly any data about the prevalence of colorectal neoplasms in symptomatic patients who are younger than 55 years of age. This study provides for the first time age-matched and sex-matched prospective data from symptomatic patients comparing total colonoscopy in symptomatic patients younger and older than 55 years. The median age of undergoing a colonoscopy due to abdominal symptoms in our screening cohort was 43 years for women and 44 years for men. A total of 864 (55.6%) women and 689 (44.4%) men underwent a colonoscopy.

In the prospective study from I. Blumenstein et al., it was shown that the detection rate of CRC was significantly equivalent in symptomatic and asymptomatic patients older than 55 years [13].

In the present study, we demonstrate that there are significant more polyps in the control cohort of symptomatic patients of at least 55 years of age (*p*<0.0001; 26.6% vs. 12.5%). Also, colorectal cancer was found significantly more often in patients older than 55 years. Age as a risk factor for CRC is a comprehensible explanation for this result. Still, six colorectal carcinomas were detected in symptomatic patients younger than 55 years of age. This result underlines the importance of undergoing a screening colonoscopy.

In about 45% of detected polyps, we found adenomas with a low-grade dysplasia. Two cases with a high-grade dysplasia were detected in women. Adenomas can degenerate, and the risk of degeneration correlates with the degree of dysplasia, the size, and the histological type [15]. Due to a risk of malignant degeneration, the detection and removal of adenomas seem to be important for the prevention of CRC [16, 17]. Due to the risk of malignant degeneration and the findings of adenomas in 45% in our screening cohort, we recommend screening colonoscopy for men and women by the age of 50.

In our study, we did not find any correlation between the occurrence of abdominal symptoms and the prevalence of polyps or CRC. Anorectal bleeding was the only significant indicator (*p*=0.03) but with only low sensitivity (44%).

The study by I. Blumenstein showed that the prevalence of polyps in symptomatic patients was significantly lower than in the asymptomatic screening cohort [13]. In a study by M. Kwak, which compared diagnostic colonoscopic findings in symptomatic young (18 until 49 years) patients from South Korea and the USA, it is demonstrated in a multivariate analysis lower gastrointestinal symptoms were not associated with the risk of any type of advanced neoplasia in young Korean patients [18]. Other large systematic reviews which evaluated several symptoms for the presence of CRC could also not find any alarm symptoms [19, 20].

In our opinion, our results also underline the importance of screening examinations, independently of symptomatic and asymptomatic patients. Due to the fact anorectal bleeding might be a significant indicator for the appearance of polyps and adenomas, further prospective studies are needed for confirmation.

Due to a higher risk of the incidence of colorectal cancer and the higher occurrence of adenomas in men than in women [21–23], Germany offers screening colonoscopy for men by the age of 50 since 2019. Women are still offered screening colonoscopies by the age of 55.

In our cohort, there is no significant difference between men and women, neither in the prevalence of polyps or in the prevalence of colorectal cancer. Polyps were removed in 14% in men and in 11% in women (*p*=0.11). CRC was detected in 0.4% of our symptomatic patient cohort. Also, there was no significant difference in the occurrence of adenomas between men and women. In a long-term prospective study by Click et al. from 2018, they examined 15,935 patients who underwent colonoscopy after an abnormal flexible sigmoidoscopy. They demonstrated that any identified adenomas were more likely to be found in men [22]. In a cross-sectional study from asymptomatic patients in Korea, 19,372 patients undergoing a screening colonoscopy were evaluated. The prevalence of adenomas and advance adenomas was higher in men than in women [24]. Some limitations from the Korean study are worth noting: the age of participating patients was between 20 and 80 years. The highest risk for the prevalence of adenomas was noticed in patients older than 50 years of age [24]. In our study, we only investigated patients under 55 years of age. Due to no significant differences in histological findings in men compared with women, we recommend screening colonoscopy also for women by the age of 50.

Interpretation of our data might be limited by the fact that we did not examine potential confounders for undergoing a colonoscopy, for example level of education or lifestyle. We matched age and sex in a defined local area in Germany. We tried to reduce this limitation by excluding patients with an increased risk for CRC (for example patients who were in surveillance after resection of CRC, diagnosed inflammatory bowel disease, familial adenomatous polyposis, hereditary nonpolyposis colon cancer, or positive family history). Another limitation is that not all histological data by the removed polyps could be transmitted to us. In total, we received 103 histological results by removed polyps from around 95 patients (out of 164 patients). In our opinion, this amount of data is still representative.

In summary, this study clearly underlines the importance of undergoing screening colonoscopies, independently of symptomatic or asymptomatic patients. Due to no significant differences between men and women in our study, screening colonoscopies should also be recommended for women by the age of 50.
